# Long-term health consequences of violence exposure in adolescence: a 26–year prospective study

**DOI:** 10.1186/1471-2458-12-411

**Published:** 2012-06-07

**Authors:** Niclas Olofsson, Kent Lindqvist, Benjamin A Shaw, Ingela Danielsson

**Affiliations:** 1 Department of Medical and Health Sciences, Division of Community Medicine, Social Medicine and Public Health Science, Linköping University, Linköping, Sweden; 2 Department of Health Policy, Management and Behavior, University at Albany, SUNY, Albany, NY, USA; 3 Department of Clinical Sciences, Obstetrics and Gynecology, Umeå University, Sundsvall, Sweden; 4 Department of Research and Development, Sundsvall Hospital, Sundsvall, Sweden; 5 County Council of Västernorrland, 871 85, Härnösand, Sweden

## Abstract

**Background:**

Violence victimization represents a serious risk factor for health related symptoms, for both men and women. The aim of this study was to evaluate the long-term effects of violence exposure in late adolescence and early adulthood on adult health, physical as well as mental, using a long-term prospective population-based study, with a follow up of 9, 19, and 26 years.

**Methods:**

The primary data source is a longitudinal panel from one of the longest running social science surveys in the world, the Swedish Level-of-Living surveys (LNU). We analyzed three cohorts, individuals aged 15–19 in 1974 and 1981, and individuals aged 18–19 in 1991 which were followed up 2000. Structured interviews on childhood, family relationships, life-events, living conditions, health history and status, working conditions, behavioral, psychosocial, and demographic variables were repeatedly used in all cohorts.

**Results:**

Multivariate models of violence exposures in adolescence in the 1974–91 cohorts as predictors of adult health in 2000 are reported for both men and women. Women exposed to violence had raised odds ratios for ill health, measured as heavy illness burden, and poor self rated health, after controlling for possible confounders. No such associations were found for men.

**Conclusions:**

This study’s findings provide additional empirical support for the importance of policies and practices to identify and prevent violence exposure in adolescence and young adulthood and to supply treatments for adolescence exposed to violence and above all the young women.

## Background

Violence victimization appears to represent a serious risk factor for health related symptoms, both in men and women and in all stages of life [1-6]. Research evidence has emerged that highlights the long-term effects of violence exposure in early life on adult health, physical as well as mental [7-11].

Life course epidemiology conceptualizes determinants of disease occurrence in terms of biological and social exposures experienced during different stages of life. It is possible that hazardous exposures throughout the life course accumulate and, thus gradually increase the risk of poor health [12]. Alternatively, the timing of an exposure could be an important factor in determining its level of long-term risk. For example, early life exposures to social and economical disadvantage could be particularly damaging in that they could increase the risk of unhealthy life trajectories; that is, early life events and environments may negatively influence later experiences, opportunities and health risk factors [13]. Additionally, adverse childhood experiences during critical periods may have latent effects that independently lead to negative adult health outcomes [14-17].

Consistent with this view of the importance of exposure timing, violence experienced during childhood and adolescence may be particularly damaging to health over time. This is because childhood and adolescence are the periods in which important personal and psychological resources that guide cognition and decision-making, and ultimately influence health, are typically developed [16,18]. Exposure to interpersonal violence could be particularly disruptive to normal psychological development when it occurs during these periods [19], whereas violence experienced at other stages of life might ultimately have relatively fewer life course consequences [20].

Previous research has in retrospective studies made significant contribution to our understanding of the lasting effects of abuse in early life [21-24]. However, to our knowledge no studies have considered the long-term health consequences of violence exposure in adolescence prospectively. The data generated from prospective studies is often considered stronger than data from cross-sectional and retrospective studies, largely because of the possibility to control for confounding variables. Prospective studies also reduce problems associated with recall bias, because subjects are not required to think back over long periods of time.

The aim of this study was to evaluate the association between adolescent exposure to violence and adult health in a long-term prospective population based study, with a follow up of 9, 19, and 26 years. Our hypothesis was that individuals who reported exposure to violence during the transition from adolescence to young adulthood were at increased risk for poor health in adulthood compared to those not exposed to violence.

## Method

### Survey design

The primary data source is the longitudinal panel from the Swedish Level-of-Living surveys (LNU), one of the longest running longitudinal social science surveys in the world. The first LNU survey was conducted in 1968, based on face-to-face interviews with a representative sample of the Swedish population aged 15–75; the lower age bracket was later changed to 18 (1991). Follow-up surveys have since been conducted at somewhat irregular intervals in 1974, 1981, 1991, 2000, and 2010 [25]. This study used data from 1974–2000 and excluded the first wave of data collected in 1968. In the first survey wave in 1968, a random sample of approximately one per 1000 of the Swedish population aged 15–75 was interviewed. In subsequent waves a new sample was included and the individuals in the original sample were retained as long as they were 75 years old or younger. This means that approximately 6500–6800 individuals were included in the gross sample sizes each survey year since the original selection sample. The response rates have varied between 90,8 percentage in 1968 to 76,6 percentage in the year 2000. Comprehensive structured interviews, guided by a checklist, on childhood, family relationships, life-events, living conditions, health history and status, working conditions, behavioral, psychosocial, and demographic variables were repeatedly used in the successive surveys [26].

### Sample

**Figure 1:**
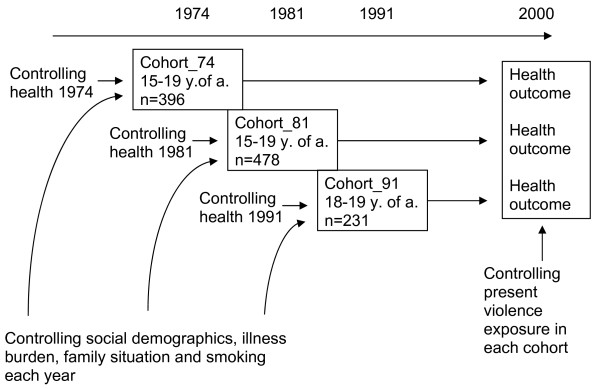
Analysis design of the samples.

We restricted our analyses to comparing three cohorts: individuals aged 15–19 in 1974 and 1981, and individuals aged 18–19 in 1991 Young men and women aged 18–19 from the 2000 survey were included to describe the social demographics at the time of ending the study, to demonstrate changes over time. The first survey wave in 1968 did not include questions about violence and was not included in the analyses. The first three cohorts were followed up through 2000, when they were 41–45 years of age, 34–38 years of age, and 27–28 years of age (Figure 1). In 2000 the health of the men and women in the cohorts exposed to violence in late adolescence (15–19, 18–19) was compared to health of the men and women from the same cohorts not exposed to violence. Of the 191 young men and 205 young women included in the 1974 cohort, 132 (69%) men and 156 (76%) women remained in 2000. Additionally, 247 young men and 231 young women in the 1981 cohort generated 185 (75%) responding men and 177 (77%) responding women in 2000. Of the 1991 cohort that included 120 young men and 111 young women, 99 (82%) men and 85 (77%) women remained in 2000 (Table 1).

**Table 1:** Social demographic descriptives, illness burden and smoking in adolescents exposed and not exposed to violence during the past year each survey year and gender (percentage with 95% CI)

	**1974**				**1981**				**1991**				**2000**			
	Men		Women		Men		Women		Men		Women		Men		Women	
	Exposed	Not exposed	Exposed	Not Exposed	Exposed	Not exposed	Exposed	Not Exposed	Exposed	Not exposed	Exposed	Not Exposed	Exposed	Not exposed	Exposed	Not Exposed
	(n = 26)	(n = 165)	(n = 19)	(n = 186)	(n = 34)	(n = 213)	(n = 14)	(n = 217)	(n = 32)	(n = 88)	(n = 11)	(n = 100)	(n = 14)	(n = 100)	(n = 11)	(n = 77)
Fathers social class^1^	46	44	42	49	68	52	64	46	47	43	46	49	23	14	36	22
	(27–65)	(36–52)	(20–64)	(42–56)	(52–84)	(45–59)	(39–89)	(39–53)	(30–64)	(33–53)	(17–75)	(39–59)	(10–26)	(0–32)	(8–64)	(13–31)
Childhood economical problem^2^	13	4	16	12	16	6	**50**	6	19	4	**36**	5	19	10	25	13
	(1–26)	(1–7)	(0–32)	(7–17)	(4–28)	(3–9)	**(24–76)**	(3–9)	(5–33)	(0–8)	**(9–64)**	(1–9)	(11–27)	(0–26)	(0–51)	(5–21)
Childhood serious illness in the family^3^	15	13	21	14	13	12	7	11	16	8	36	17	Na	Na	Na	Na
	(1–29)	(8-18	(3–39)	(9–19)	(2–24)	(8–16)	(0–20)	(7–15)	(3–29)	(2–14)	(8–64)	(10–24)				
Childhood family status^4^	77	87	74	89	74	83	64	83	81	77	63	72	81	60	67	64
	(61–93)	(82–92)	(54–94)	(84–93)	(59–89)	(78–88)	(39–89)	(78–88)	(67–95)	(68–86)	(34–92)	(63–81)	(73–89)	(34–86)	(39–95)	(53–75)
Education IP^5^	0	6	5	4	10	7	14	9	9	2	0	7	34	57	27	42
	(0–0)	(2–10)	(0–15)	(1–7)	(0–20)	(4–10)	(0–32)	(5–13)	(0–19)	(0–5)	(0–0)	(2–12)	(25–43)	(31–83)	(1–53)	(31–53)
Illness burden IP^6^	**46**	21	53	46	32	14	**86**	33	28	22	**82**	41	45	40	58	44
	**(27–65)**	(15–27)	(31–75)	(39–53)	(16–48)	(9–19)	**(68–99)**	(27–39)	(12–44)	(13–31)	**(59–99)**	(31–51)	(35–55)	(14–66)	(29–87)	(33–55)
Smoking IP^7^	42	30	68	47	36	19	**71**	31	28	14	18	24	20	13	33	18
	(23–61)	(23–37)	(47–89)	(40–54)	(20–52)	(14–24)	**(57–95)**	(25–37)	(12–44)	(7–21)	(0–41)	(16–32)	(0–41)	(6–20)	(5–61)	(9–27)
Percentage of IP at follow up 2000	62	70	58	78	74	75	71	77	84	82	91	75				
	(43–81)	(63–77)	(36–80)	(72–84)	(59–89)	(69–81)	(47–95)	(71–83)	(74–94)	(74–90)	(74–100)	(67–83)				

^1^Social group III; ^2^ Yes; ^3^Yes; ^4^ Living with both parents; ^5^ Senior high school or above; ^6^ Heavy illness burden; ^7^ Smoking;

*IP* Interview person; *Na* not applicable because of missing data; Bold figures = significant differences between exposed and not exposed.

### Measures

Exposure to violence was measured in 1974, 1981, 1991, and 2000 using the following three questions: ‘In the last twelve months, have you been exposed to any of the following? 1) ‘Violence causing visible marks or injury? 2) Violence not causing visible marks or injury? 3) Threat or threats that were dangerous or serious enough to frighten you?’ Respondents answered ‘Yes’ or ‘No’ to each question. Responses to these three questions were combined and dichotomized such that individuals exposed to any form of physical violence or threats were considered to be exposed to violence and distinguished from individuals who were not exposed to violence.

Questions tapping health were also measured in 1974, 1981, 1991, and 2000. The main health outcome measure was constructed from a long list of symptoms, signs of disease and manifest diseases, introduced by the question ‘During the past 12 months, have you had any of the following illnesses or ailments?’ For each item the response alternatives were ‘No’, ‘Yes, minor problems’, and ‘Yes, severe problems’. The list was comprised of different kinds of health status information (such as coughing, vomiting, chest pain, gall bladder problems, nervous troubles, high blood pressure, diabetes, or cancer), including symptoms and feelings as experienced by the interviewee directly (e.g., chest or stomach pain, dizziness), as well as test results and diagnoses obtained from a physician (e.g., anemia, bronchitis or diabetes) [26]. In this study we used the list of symptoms and diseases to capture the burden of ill health in total, which has been used in several previous studies. An index of forty-two items, included in all survey waves, was used capturing those “free of health problems” (score 0–5) and those with “a heavy illness burden” (score 6 or more) [27-29].

Another outcome of interest was the respondents self rated health (SRH), measured with the question “How would you rate your health”. The response alternatives were “Good”, “In between” or “Bad”. In the analyses, SRH was dichotomized into “Good” versus “Bad” or “In between”. In a number of studies, this question of self-rated health has been found to be an excellent predictor of future health [28,30].

Questions about social demographics, behavioral, familial and economical characteristics were included in the 1974, 1981, 1991, and 2000 surveys. Social class of origin was based on main occupation of the father. Using the Swedish socioeconomic classification [31] as the basis for measuring social class, a three-level variable was coded (social class I to III). In the analyses a dichotomization was used, social class I and II, verses III, this essentially equates to non-manual work verses manual. In addition to social class of origin, the following indicator of childhood hardship was included; “Did your family suffer from economic difficulties during your upbringing”. The response alternatives were “Yes” or “No”. Despite the simplicity of this indicator, evidence of its importance for adult health status is strong [27]. Severe illness in the family was covered with the question “Was any member of your immediate family afflicted with serious or prolonged illness during your upbringing”. A dichotomy “Yes” or “No” was used in the analyses. A behavioral characteristic, daily smoking was measured by asking respondents whether or not he/she currently smoked. Response options included: “Yes, but less than 10 cigarettes per day or the equivalent”, “Yes, 10 or more cigarettes per day or the equivalent”, and “No”. Responses were dichotomized into “Yes” or “No”. The respondents’ educational level was used in a dichotomized form, distinguishing between those who finished senior high school education and those who did not. Childhood family status was measured using the following question. “Did you live with both your natural (biological) parents during your whole upbringing?” The response alternatives were yes and no; if no: parents divorced, parent/s’ dead, or parent absent. In the analyses a dichotomy between “Yes” or “No”, was used.

An application for permission to use the data was sent to Swedish National Data service (Obligation 081114 Svensk Nationell Datatjänst, SND). SND did an ethical assessment along with a judgement of the research plan before allowing the researchers access to the data. Permission has also been received by the original authors.

### Statistical analyses

**Figure 2:**
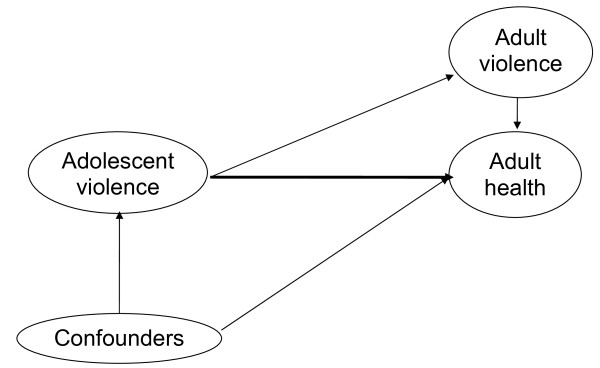
Model to be tested.

In order to assess the independent association between being exposed to violence in adolescence and adult health, the analyses controlled for potential confounders measured early in life, as well as adulthood violence exposure (see Figure 2).

The first step of the analyses was to explore the prevalence of social demographics, health outcomes and smoking in adolescent men and women exposed and not exposed to violence for each cohort, during every period (see Figure 1 and Table 1). This first step was taken to be able to describe the general societal trends in Sweden. But these analyses were also done to identify potential confounders of the relationship between violence exposure in late adolescence and adult health (see Figure 1). When trying to estimate effects over time, a potentially important consideration is time-varying confounders and/or exposures. Past and present potential confounders and exposures have to be analyzed. In order to control the existence of time-varying confounders and/or exposures the researchers have to model not only past exposures but also present exposure in order to more correctly estimate the outcome [32]. As social demographics and social mobility seem to be more unalterable and slow changing processes [33,34] than exposure to violence [35], the researchers decided to control only for present violence the follow up year 2000.

The multivariate analyses in the second step were conducted to include the potential confounders in the analyses if there was theoretical or empirical support for its potential as a risk factor to a negative health outcome. A series of multiple-predictor models estimated the impact of late adolescence violence exposure on the severe illness burden and self-rated health (SRH) outcomes. Different models accounting for various potential confounders of the observation between exposure to violence and health were estimated.

A Hosmer-Lemeshow test (H-L goodness of fit test) test was done to give an indication of the fit of the different multivariate models. Nagelkerke R^2^ was estimated for each multivariate model to give an indication of the explaining value of the adjusted models. All statistical analyses were performed by using SPSS 19.

## Results

In Table 1 the different age cohorts´ social demographic characteristics in relation to violence exposure in the last year at the time of each survey are described. Few significant differences were seen between exposed and unexposed men and women. But there were tendencies in the 2000 cohort compared to the 1974, 1981 and 1991 cohorts towards fewer manual working fathers, higher educational level and fewer smokers, both among the exposed and not exposed young men and women. Also there was a tendency, at least in the non exposed group, towards a lower likelihood of living with both parents (for example chronologically 87%, 83%, 77%, and 60% among the men vs. 89%, 83%, 72% and 64% among the women). Childhood economic problems were significantly more common in young women exposed to violence from the 1981 cohort (see Table 1). The father’s social class, childhood severe illness in the family, education and childhood family status did not differ significantly between the exposed and not exposed young men and women in these samples. They have been used as confounders/predictors to ill health in other studies, which qualified them to be used in the further multivariate analyses [13]. Education though, was excluded because of no empirical support and no basis from earlier studies.

Illness burden and daily smoking at the first interview are also reported in Table 1. Both variables showed some statistically significant differences between those reporting violence exposure compared to those not being violence exposed. These results indicate that health differences in the cohorts were present, and had to be controlled for in the further analyses, when regressing different cohort exposures of violence (1974, 1981, and 1991) against the health outcome 2000.

In Table 2 the multivariate model of violence exposure in adolescence in the 1974 cohort and adult health 2000 is reported for men and women. Compared to the unexposed, women exposed to violence in 1974 had elevated odds for heavy illness burden (5.2 (1.0–28)) as well as bad SRH (6.3 (1.6–25)) in 2000, after controlling for possible confounders; similar findings were not evident among men. The same trends were seen in the 1981 cohort (Table 3) as well as in the 1991 cohort (Table 4). In the 1981 cohort (Table 3), women exposed to violence during the past year had increased odds of heavy illness burden in 2000 (4.5; (1.2–17)), but violence exposure in adolescence was no longer associated with current illness burden after controlling for recent violence exposure.

**Table 2:** Multivariate model of predictors of heavy illness burden and bad self reported health (SRH) over time (1974 to 2000), in men and women who have reported versus not reported violence exposure with adjustment for risk factors for poor health (Odds ratio with 95% confidence interval)

	**Heavy illness burden 2000**				**Bad SRH 2000**			
	Unadjusted model 1		Adjusted model 2^1^		Unadjusted model 1		Adjusted model 2^1^	
	Men	Women	Men	Women	Men	Women	Men	Women
Reported violence IP 1974								
No	1	**1**	1	**1**	1	**1**	1	**1**
Yes	1.4 (0.5–4.2)	**2.8 (1.7–11)**	1.1 (0.4–3.6)	**5.2 (1.0–28)**	1.2 (0.4–4.2)	**6.7 (1.8–24)**	1.1 (0.3–3.9)	**6.3 (1.6–25)**
Fathers social class								
I and II			1	1			1	1
III			1.8 (0.9–4.0)	0.8 (0.4–1.7)			1.5 (0.6–3.4)	1.6 (0.7–3.6)
Childhood economical problem								
No			1	**1**			1	1
Yes			1.7 (0.2–11)	**5.7 (1.7–19)**			0.5 (0.1–5.6)	2.0 (0.7–5.8)
Childhood health problem in family								
No			1	1			1	1
Yes			1.1 (0.4–3.3)	0.9 (0.3–2.5)			1.3 (0.5–4.2)	0.9 (0.3–2.7)
Childhood family status								
Both parent			1	1			1	1
Divorce/dead/absent			1.5 (0.5–4.8)	2.0 (0.6–6.2)			1.2 (0.3–4.3)	1.7 (0.5–5.4)
Illness burden 1974 IP								
No			**1**	1			**1**	1
Yes			**3.6 (1.5–8.6)**	1.6 (0.8–3.2)			**2.7 (1.1–7.0)**	1.0 (0.4–2.3)
Smoking 1974 IP								
No			1	1			1	1
Yes			1.0 (0.4–2.3)	0.8 (0.4–1.6)			0.6 (0.2–1.7)	1.2 (0.5–2.7)
Reported violence IP 2000								
No			1	1			1	1
Yes			0.6 (0.1–5.4)	1.5 (0.4–5.2)			1.0 (0.1–9.4)	1.6 (0.4–6.0)

Nagelkerke R^2^ for the adjusted model heavy illness burden (men 0.14 women 0.15) and the adjusted model bad SHR (men 0.07 women 0.11).

**Table 3:** Multivariate model of predictors of heavy illness burden and bad self reported health (SRH) over time (1981 to 2000), in men and women who have reported versus not reported violence exposure with adjustment for risk factors for poor health (Odds ratio with 95% confidence interval)

	**Heavy illness burden 2000**				**Bad SRH 2000**			
	Unadjusted model 1		Adjusted model 2^1^		Unadjusted model 1		Adjusted model 2^1^	
	Men	Women	Men	Women	Men	Women	Men	Women
Reported violence IP 1981								
No	1	**1**	1	1	1	**1**	1	**1**
Yes	0.4 (0.4–2.8)	**9.0 (1.1–33)**	0.8 (0.3–2.1)	2.0 (0.8–31)	2.5 (0.9–6.7)	**4.8 (1.3–18)**	2.6 (0.8–8.3)	**7.3 (1.1–46)**
Fathers social class								
I and II			1	1			1	1
III			1.4 (0.7–2.8)	1.3 (0.7–2.5)			0.7 (0.3–1.7)	1.3 (0.6–3.0)
Childhood economical problem								
No			1	1			1	1
Yes			0.5 (0.1–2.9)	1.3 (0.3–6.2)			2.9 (0.4–19)	0.3 (0.1–2.0)
Childhood health problem in family								
No			1	1			1	1
Yes			2.0 (0.8–5.0)	0.9 (0.3–2.6)			1.9 (0.6–5.9)	1.1 (0.3–4.0)
Childhood family status								
Both parent			1	1			1	1
Divorced, dead or absent			1.4 (0.6–3.6)	0.8 (0.7–4.7)			0.6 (0.2–2.3)	2.2 (0.8–6.0)
Illness burden 1981 IP								
No			**1**	1			1	1
Yes			**2.2 (1.0–5.4)**	1.3 (0.7–2.7)			0.3 (0.1–1.2)	1.7 (0.7–4.1)
Smoking 1981 IP								
No			1	1			**1**	1
Yes			1.2 (0.5–2.6)	0.8 (0.4–1.7)			**2.9 (1.2–7.4)**	1.7 (0.7–4.0)
Reported violence IP 2000								
No			1	**1**			1	1
Yes			1.5 (0.4–6.4)	**4.5 (1.2–17)**			4.2 (0.9–20)	0.6 (0.1–2.5)

Nagelkerke R^2^ for the adjusted model heavy illness burden (men 0.08 women 0.15) and the adjusted model bad SHR (men 0.07 women 0.10).

**Table 4:** Multivariate model of predictors of heavy illness burden and bad self reported health (SRH) over time (1991 to 2000), in men and women who have reported versus not reported violence exposure with adjustment for risk factors for poor health (Odds ratio with 95% confidence interval)

	**Heavy illness burden 2000**				**Bad SRH 2000**			
	Unadjusted model 1		Adjusted model 2^1^		Unadjusted model 1		Adjusted model 2^1^	
	Men	Women	Men	Women	Men	Women	Men	Women
Reported violence IP 1991								
No	1	**1**	1	**1**	1	**1**	1	**1**
Yes	1.6 (0.7–3.9)	**3.1 (1.8–13)**	1.3 (0.5–3.4)	**2.1 (1.0–11)**	0.9 (0.1–8.3)	**3.4 (1.1–10)**	0.5 (0.1–6.3)	**3.2 (1.0–11)**
Fathers social class								
I and II			1	1			1	1
III			1.2 (0.5–3.0)	0.4 (0.1–1.0)			1.4 (0.4–4.6)	0.5 (0.2–2.2)
Childhood economical problem								
No			1	1			1	1
Yes			8.7 (0.9–44)	2.0 (0.3–14)			4.2 (0.6–27)	3.3 (0.6–33)
Childhood health problem in family								
No			1	1			1	1
Yes			0.6 (0.2–2.6)	1.2 (0.3–4.8)			0.9 (0.1–11)	0.5 (0.1–5.5)
Childhood family status								
Both parent			1	**1**			1	1
Divorced, dead or absent			0.7 (0.2–2.6)	**3.2 (1.1–10)**			0.6 (0.1–4.0)	1.1 (0.2–6.2)
Illness burden 1991 IP								
No			1	1			1	1
Yes			2.5 (0.9–7.5)	1.3 (0.5–3.3)			1.7 (0.5–7.0)	2.4 (0.5–12)
Smoking 1991 IP								
No			1	1			1	1
Yes			1.3 (0.4–4.5)	1.3 (0.5–3.7)			1.7 (0.3–7.6)	1.6 (0.4–9.1)
Reported violence IP 2000								
No			1	1			1	1
Yes			1.1 (0.2–5.7)	1.8 (0.4–7.1)			1.9 (0.2–17)	1.2 (0.2–9.3)

Nagelkerke R^2^for the adjusted model heavy illness burden (men 0.10 women 0.11) and the adjusted model bad SHR (men 0.13 women 0.17).

For men in the 1974 and 1981 cohorts, being exposed to violence in adolescence was not associated with future health problems, but having a heavy illness burden during the survey years 1974 and 1981 (Table 2 and Table 3) was associated to increased odds ratios of heavy illness burden 2000 (1974; 3.6 (1.5–8.6)) in Table 2 and (1981; 2.2 (1.0–5.4)) in Table 3. Heavy illness burden in the survey years 1974 and 1981 also increased the odds of bad SRH in 2000 (2.7 (1.1–7.0) 1974; Table 2 and 2.9 (1.2–7.4) 1981; in Table 3) among men.

The Hosmer-Lemeshow test implied that all the multivariate models’ (Table 2-Table 4) estimates fit the data at an acceptable level [36]. The Nagelkerke R^2^ show moderate effect sizes which indicate that the multivariate adjusted models in comparison with the unadjusted models are better explaining models.

## Discussion

In this long-term prospective study, young women exposed to violence in late adolescence had increased odds of heavy illness burden and bad self reported health in adulthood compared to non-exposed women, controlling for social demographics, health and smoking and adult violence exposure. The men did not show the same relationship between violence exposure in adolescence and increased odds of heavy illness burden or bad self-reported health.

Research has shown in several important respects that there is a cross-sectional and retrospective relationship between violence exposure and negative health outcomes [1-3,22]. Few, if any, prospective studies showing long-term relationships between exposure to violence and adverse health have been published. The objective of most studies is to prove a casual relationship between two variables; that is, a change in one variable "causes" a change in the other, rather than an associative relationship. An associative relationship is not necessarily causal, but can be explained by the presence of other 'un-seen' variables to which the two variables being studied are themselves separately linked. Prospective studies are often regarded as strong as they deal methodologically with difficulties such as confounding and other biases. This study suggests that exposure to violence in young women may have a longitudinal relationship to negative health outcomes. The men did not show a similar distinct relationship. Instead, poor health status in earlier life was more strongly related to negative health outcomes in the long-term.

Lately, two emerging understandings of how early experiences of violence may affect adult health have been established; (1) latent effects of adversities during critical periods and (2) accumulated exposure of stressful experiences [37]. The first theory is explained by the existing evidence that suggests that early childhood trauma (including violence exposure, abuse and neglect) activates stress associated hormonal and neurochemical systems in the body that under normal circumstances are protective but become toxic with severe exposure, with resulting negative physical effects on the body [37-39].

The second theory is built on the strong relationship between retrospective adult reports of traumatic childhood or adolescent incidents and/or amount of reported violent episodes and increased prevalence of health impairments in adulthood [21-23]. In relation to accumulating traumatic childhood or adolescent events, family characteristics (such as parental psychopathology, parental loss or absence or parental divorce) during the upbringing contribute to the development of subsequent future health-related well-being or problems in adulthood [40,41]. Also, persons who have experienced adversities during their upbringing, are more likely to participate in high-risk behaviors [41,42], which are related to both negative health and violence [43]. Continual psychological pressure and/or persistent wear and tear of the body due to repeated stressful or traumatic experiences over the life course might dysregulate the normal physiological adaptations to stress and threats, and later sensitivity to stress [37,39], or influence immune functioning which may in turn contribute to increased adult health problems [44].

Any of these theories may explain the long-term effects on health seen in our study. It is reasonable to assume that the violence exposure in the life stage of adolescence, as well as in childhood, exercise negative long term effects on health [20,21,45,46], while several crucial developmental psychological transitions are negotiated, in relation to other stages in life [20,21,46]. Also, we do not know the amount of possible accumulating adverse events, but it is well-known that previous exposure to violence is a strong risk factor for further exposure [47-49].

In our study the results also express distinct gender differences concerning the prospective effects of reported exposure to violence in adolescence and health status in adulthood. This has been demonstrated in one earlier short-term prospective study [50] and in some cross-sectional studies [50,51], Several reasons for this have been put forward. The magnitude, nature and health impact of violence differ greatly for young men and women. In a study by Sundaram et.al. 2004, young men were significantly more likely to experience violence than women [50,52] but the associations between physical violence, poor self rated health and self reported morbidity were significant for women, but not men. Danielsson et. al. (2009) showed in their study pronounced gender differences in adolescent and young adults, both in type, prevalence and outcomes of exposure to violence [51]. The young women reported more severe adverse effects from all types of abuse than the men. It is probable that gender specific experiences of violence and gender differences in health perceptions interact and contribute to a gender specific process of victimization [47].

Gender differences in health outcomes could also be understood as having possible biological explanations [53]. Research has shown sex differences in brain maturation during childhood and adolescence indicating possible diverse developmental pathways due to different or similar adverse experiences such as violence exposure [50,53-55]. One potential mechanism is sex differences in the development of brain structures that process experiences (HPA axis; hypothalamic-pituitary-adrenal axis). In females, there is an increased response of the HPA axis to stress with advancing puberty, while in males the response is decreased, possibly associated with increased testosterone levels [39,56]. This, in connection to the stress associated with violence exposure, might differentiate males and females with respect to the rates of onsets, courses and symptomatology of common psychiatric disorders and psychological symptoms [56]. It is well known that women in the general population in all ages, have higher rates of post traumatic stress disorder (PTSD), which could indicate a psychological fragility were violence exposure could be more harmful to young women than young men [57,58]. Furthermore; adolescence has been described as the identity formation life stage [59]. Many factors may play a role in this period of life, including different stresses associated with social gender expectations related to men and women gender roles, the higher incidence of exposure to trauma experiences in young females, and differences in social cognitive function such as rejection sensitivity, or even a gendered difference in reporting symptoms [56,58,60].

### Life course remarks

In order to isolate a potential link between adolescent exposure to violence and adult health, societal changes have to be looked upon and possibly controlled for [61,62]. In our samples, general patterns are apparent, but these patterns were generally not statistically significant; the educational level rose, and the percentage of social class III families decreased from the 1974 to the 2000 cohorts, and so did smoking. On the other hand, the percentages of reported childhood economical problems were almost steady throughout the entire research cohort era. Also, there was a tendency for two parent families to decrease during the same period.

As three different survey year cohorts (1974, 1981, and 1991) are studied in relation to the 2000 health outcome, four different historical contexts are possible to reflect on. During the studied period 1974–2000, a number of significant changes did take place in the Swedish society. In welfare terms, the study period begins when the Swedish welfare state was still expanding and ends during a period of retrenchment. The labor market situation deteriorated from almost full employment to high unemployment [61,63]. Although there have been a changing historical context during the whole study period, there has not been an economical collapse with depression and familial deprivation as a consequence. But understanding and controlling the historical context within the performed studies make it more possible to rule out potential societal effects over time and allow the focus to be on the primary exposures and outcomes.

### Study limitations

This study has certain limitations. First, the relative low sample size affects the power of the results. The sample size together with some low frequencies could generate numerical problems and introducing wide confidence intervals [64]. Despite this, significant results were found. Still, an increased chance of false negatives remains, for example with regard to the low number of statistical significant differences found among the social demographic descriptives (Table 1). Second, the measures used in this study to capture exposure to violence are crude and possibly underestimate the prevalence of exposure violence. Third, it is possible that respondents’ conceptual understandings of some survey questions might have changed throughout the study period. For example, the importance of different health related assessments varies between adolescence and adulthood since health problems differ between adolescence and adulthood (the study is framed in a broad age range), and probably between the earlier survey cohort and the later [65]. However, in our study even after controlling for family upbringing related factors, behavioral factors, and adolescent illness burden, there remained a significant relationship between adolescent exposure to violence and adult health status of the women.

## Conclusions

After controlling for family upbringing related factors, familial economical situation, behavioral factors, and adolescent illness burden, there remained a significant relationship between adolescent exposure to violence and adult health status measured as illness burden and self-reported health of women. In contrast, men’s adult illness burden and self-reported health seemed to have been affected by illness burden in adolescence rather than exposure to violence in late adolescence. Having prospectively shown a probable relationship between adolescent exposure to violence and negative general health status in adulthood, measured by illness burden and self-reported health, the next step would be to disentangle the severe illness burden. Severe symptoms, but also specific illnesses and diseases, should be identified to help us to better understand the nature of the long-term effects of violence exposure.

## Competing interests

The authors declare that they have no competing interests.

## Authors' contributions

All authors read and approved the final manuscript. NO conducted the literature search, reviewed and categorized the articles and had primary responsibility for writing the manuscript. KL and BAS both participated in interpreting the studies results and helped revise the manuscript. ID participated in interpreting the studies results, helped revise the manuscript, provided input on the various drafts, and read and approved the final manuscript.

## Pre-publication history

The pre-publication history for this paper can be accessed here:

http://www.biomedcentral.com/1471-2458/12/411/prepub
